# Chronic headache patients’ health behavior and health service use 12 months after interdisciplinary treatment – what do they keep in their daily routines?

**DOI:** 10.1186/s12883-022-02646-w

**Published:** 2022-04-21

**Authors:** Carolin Donath, Katharina Luttenberger, Christa Geiß, Patricia Albert, Britta Fraunberger

**Affiliations:** 1 Department of Psychiatry and Psychotherapy, University Hospital Erlangen, Friedrich-Alexander-Universität Erlangen-Nürnberg, Center for Health Services Research in Medicine, Schwabachanlage 6, 91054 Erlangen, Germany; 2 Interdisciplinary Pain Center, University Hospital Erlangen, Friedrich-Alexander-Universität Erlangen-Nürnberg, Krankenhausstr. 12, 91054 Erlangen, Germany

**Keywords:** (MeSH): Headache – Headache Disorders, Primary – Headache Disorders – Pain Management – Quality Indicators, Health Care – Patient-Reported-Outcome Measures – Patient Outcome Assessment – Quality Assurance, Health Care – Health Services Research

## Abstract

**Background:**

We do not yet know whether or the extent to which multimodal therapy changes the health behaviors and health service use of chronic headache patients in the long term. Associations are expected between pain symptoms and pain management abilities for patients who are categorized as successfully treated and those who remain unchanged.

**Methods:**

Routine longitudinal data of an enrolment period of five years from 101 headache patients treated with a two-week, full-day, semi-inpatient multimodal pain therapy at the Interdisciplinary Pain Center of the University Clinic Erlangen were available when therapy began and 12 months after treatment. To investigate long-term changes in health behavior and health service use as well as their associations with the outcome “reduction in pain days,” we used descriptive and inferential statistics (i.e., binary logistic regression).

**Results:**

Patients who underwent interdisciplinary treatment showed statistically significant changes in their health behavior in five areas. Twelve months after treatment, we found a significantly higher frequency of engagement in athletic sports (*p* < .001) as well as increases in the use of relaxation techniques (*p* < .001), TENS devices for relaxation purposes (*p* = .008), psychological coping strategies (*p* < .001), and mindfulness-based techniques for dealing with pain (*p* < .001). 52.8% of the sample reported a reduction in the number of pain days 12 months after treatment. Binary logistic regression (χ^2^ (12) = 21.419; *p* = .045; *R*^*2*^ = .255) revealed that a reduction in pain days 12 months after treatment was positively associated with regular physical activity in the form of muscle strengthening and stretching (athletic sports) (*p* = .012).

**Conclusion:**

Chronic headache patients acquired long-term skills from an interdisciplinary treatment concerning the use of relaxation techniques, the use of psychological coping strategies, and physical activity in the form of athletic exercise. Of those, regular athletic exercise was positively associated with a smaller number of pain days in the long term. Thus, a physical activity module should be an element of interdisciplinary treatment for chronic headache patients.

**Supplementary Information:**

The online version contains supplementary material available at 10.1186/s12883-022-02646-w.

## Background

Pain is defined as “an unpleasant sensory and emotional experience associated with, or resembling that associated with, actual or potential tissue damage” in an updated definition by the International Association for the Study of Pain (IASP) [1, 2]. Chronic pain is defined as pain “that persists beyond the normal tissue healing time (usually approximately three months) without apparent biological value” and takes the factors durability and appropriateness into account [3, 4]. The prevalence of chronic pain is reported to lie between 10.1% and 55.2% based on systematic reviews and meta-analyses [4, 5]. However, differences in the use of definitions of chronic pain were evident. A subcategory of pain is headache. The forms of headache most often reported are migraines and tension-type headaches [6]. Following those are a medication-overuse headache that can occur in episodic or chronic forms [7]. A chronic migraine, for example, is defined as having a headache on more than 14 days in a month with at least 8 of them being a migraine [8]. According to worldwide epidemiological studies (“Global Burden of Disease”), the prevalence of tension-type headaches is 22%, and the prevalence of migraines is 15%. Thus, they are highly common conditions in the general population and are associated with a tremendous loss of quality of life and a high burden of disease [9]. According to Steiner (2015), headache disorders are in third place worldwide in the ranking of leading causes of disability computed in years of life lived with a disability (YLDs) [10]. According to Saylor and Steiner [9], migraines alone are the third most important cause of disability in the age group of 15–49 year old. A review stated that the burden of disease for migraine patients is higher than for tension-type headache patients or non-headache patients [11]. More recent studies by the WHO-coordinated “Lifting the Burden” group have suggested even a higher prevalence of migraines (20%) than the Global Burden of Disease estimated. The Lifting the Burden group reported tension-type headaches as the most common headache subtype with a prevalence of 42% worldwide [6, 7]. Members of the Eurolight project have claimed the cost of all headache disorders to be 173 billion per year in the EU, and this rate has been officially more conservatively estimated at 112 billion [12]. The WHO stated in 2016 that headache disorders are not only underestimated but also undertreated [13]. According to the WHO as well as to an Italian epidemiological study, about half of all headache patients try to treat their disorder themselves [13, 14].

There are different therapeutic approaches for treating headaches. First line treatment is pharmacotherapy based on principal national and international guidelines and consists of acute and preventive medications [15–17]. For acute treatment of tension-type-headache and migraine aspirin and NSAIDs are effective. Additional substances like triptanes and antiemetic drugs are available for more severe migraine attacks. Preventive therapy is used daily and should be considered for patients with high economic, physical and social impact of headache. Preventive migraine agents for example are metoprolol, topiramate and tricyclic antidepressants. Onabotulinum toxin A is an option for patients with chronic migraines. The injections are performed quarterly. A new treatment option based on migraine pathology is CGRP as target. Since 2019 three CGRP monoclonal antibodies are available for the prevention of migraine in Germany [18]. For tension-type-headache only tricyclic antidepressants are available for preventive purpose [15]. On the other hand, there are more complex intervention, combining pharmacotherapy and non-pharmacological therapeutic actions, such as interdisciplinary treatment. Existing guidelines differentiate the treatment and prophylaxes of pain according to the headache type [15, 16, 19]. For example, in chronic migraine with severe attacks a pharmacological prophylaxis together with behavior modification should be offered. The therapy of it should be pharmacological as well as accompanied from relaxation techniques, behavioral measures of stress management, teaching of coping strategies with pain and suggests regular exercise [16]. The latter named non-pharmacological modules are elements together with pharmacotherapy of interdisciplinary treatment. The IASP defines interdisciplinary treatment as a “multimodal treatment provided by a multidisciplinary team collaborating in assessment and treatment using a shared biopsychosocial model and goals” [1].

The question is whether such interdisciplinary treatments, with proven effectiveness [20–22], can lead to changes in long-term health behavior and the use of health services in treated headache patients. Interdisciplinary treatment has to be located at “Level 3” in the suggested three-tier system by the European Headache Federation and the Lift the Burden group. Level 3 consists of providers with fully trained specialists that should manage 1–2% of all headache cases, concentrating on those with highly complex primary headache disorders along with a full range of secondary headache disorders [9]. Thus, from a health-economic perspective, it would be desirable that patients with a complex headache disorder who are treated with a rare, intensive, multimodal therapeutic approach [23] are able to keep up the strategies and skills they acquired in a timely intensive intervention. This is especially true as it has become apparent that a changed health behavior—including an implemented routine of aerobic exercise and relaxation techniques along with pharmacological prophylaxis—is beneficial and preventive for chronic headache patients [24]. There is a research gap concerning the health behavior and the health service use of headache patients treated with an interdisciplinary treatment. Only one representative study was conducted in Germany involving physicians’ use of headache patients in the general population [25]. To our knowledge, health-service utilization data and the health behavior status of headache patients receiving an interdisciplinary treatment have yet to be studied. Thus, in a consecutive sample of headache patients who received an interdisciplinary treatment, the aims of this study are:

1. To explore the status and changes in the health behavior and health service use of the patients one year after treatment.
2. To investigate which health behaviors/skills taught in interdisciplinary therapy and which uses of outpatient medical and non-medical care are associated with the successful treatment of headache symptoms.

The above mentioned aims led to the following research questions:

1. Did the health behavior and the use of health services in the sample of headache patients who received interdisciplinary treatment change significantly from the beginning of treatment to one year after treatment?
2. Are certain health behaviors or patterns in the use of health services in the year after treatment associated with a successful treatment based on a reduction in the number of days patients experienced headaches one year after interdisciplinary therapy? And if so, which of them?

## Methods

### Design

Routine longitudinal data collected from chronic headache patients treated with semi-inpatient interdisciplinary group therapy in 2015 – 2020 at the Interdisciplinary Pain Center of the University Clinic Erlangen were assessed. The patients took part in a multimodal headache-specific interdisciplinary pain therapy that has been evaluated before [26]. Participants were assessed by trained pain therapists and with self-reported questionnaires included in the documentation routine of “t1” (start of therapy) and “t2” (12-month follow-up). The patients were informed about the kind of data that would be collected during their treatment and asked for consent. They gave written consent to be treated and to have their data collected, saved, and pseudonymously analyzed for scientific use. The data were pseudonymized and transferred into another format. The person carrying out the data analysis did not see any person-related information in the data, nor did they have access to databases with person-related information. They also did not see any patients in person so that it would be impossible to form a connection between sensitive data and real people. The data were treated according to the European Data Protection Legislation and its German and Bavarian administrative implementation. Routine data collection accompanies therapeutic action and fulfills documentation purposes at the University Clinic Erlangen as a matter of quality assurance. This is in accordance with the ethical commission of the Friedrich-Alexander-Universität Erlangen-Nürnberg.

### Sample

Data were collected from 14 groups during the evaluation period. 106 patients were available when therapy began, of which 101 provided follow-up information. Single missing values in the outcome variable (< 3%) were imputed with the EM algorithm. There were three cases with a missing value in one predictor variable, for which the missing values were also imputed.

The mean age of the analyzed sample was 38.1 (SD 12.6) years, and the majority were women (92.1%). Less than half were married (39.8%), and a small number were divorced (6.1%). However, only about one fourth of the sample was living alone in their household (22.9%), and the rest had cohabitants. Most of the patients did not have a migration background (89.1%). 44.9% of the participants had 10 years of education or less, whereas 37.8% had a university degree. About three quarters (74.5%) had vocational education and training. The majority of the sample was still active in the workforce or in the education track – 83.5% had contracts for paid activity. Only 17.1% had an actual certificate of illness, and 7.3% were on permanent sick leave. A minority had an accepted degree of disability (12.2%). A large proportion of the participating headache patients had been suffering from pain for at least 5 years (75.8%), 14.1% had suffered from chronic headache problems for 2 to 5 years, and only 3.0% stated that they had been suffering for less than 1 year. A total of 11.2% claimed to have permanent pain with slight deviations, 14.3% claimed to have permanent pain with large deviations, 26.5% described pain attacks with pain between the attacks as well, and the majority of the sample reported regular and frequent pain attacks but without pain between the attacks (48.0%). Patients with pain attacks usually had them several times a week (59.5%) or several times a month (29.7%). For more than half of the patients who suffered from attacks, the attacks lasted up to three days (54.1%), and for a minority (10.8%), they lasted even longer. One third of the patients who suffered from attacks had average attack durations of several hours (33.8%). At the start of therapy, patients self-classified their headaches as the following: 81.2% migraine, 69.3% tension-type headache, 7.9% other type of headache (multiple responses possible).

Patients were also classified by medical doctors evaluating their headache symptomatology and categorizing them with ICD-10-diagnoses. The IHS-classification (ICHD-3) was considered (https://ichd-3.org/de) [27]. The sample consists of 1. patients with chronic migraine (ICD-10: G43.3, G43.8, G43.9) (*n* = 17; 16.0%), 2. with episodic migraine (ICD-10: G43.0, G43.1) (*n* = 18; 17.0%) and 3. with tension type headaches (ICD-10: G44.2) (*n* = 15; 14.2%) as well as 4. patients with a combination of migraine and tension-type headache (ICD-10: G44.2 und G43.x) (n = 56; 52.8%) (all based on medical-doctors’ classified diagnoses). There, weren’t any patients with trigeminal autonomic cephalalgias (e.g. cluster headache).

Thus, according to the ICHD-3 classification the evident groups 1 and 2 refer to Part I (Primary Headaches), no. 1: https://ichd-3.org/de/1-migrane/; group 3 also refers to Part I (Primary Headaches), no. 2: https://ichd-3.org/de/2-kopfschmerz-vom-spannungstyp/; and group 4 combines both. Before therapy start a minority of the sample (*n* = 9; 8.5%) also received the diagnoses medication overuse and medication-induced headache (ICHD-3: Part II (Secondary Headaches), no. 8: https://ichd-3.org/de/8-kopfschmerz-zurueckzufuehren-auf-eine-substanz-oder-deren-entzug/).

We describe the four subgroups each with subgroup-specific frequencies concerning medication overuse: categorized were patients with 1. chronic migraine without medication overuse (*n* = 15; 88.2%) and with medication overuse (*n* = 2; 11.8%); 2. patients with episodic migraine without medication overuse (*n* = 17; 94.4%) and with medication overuse (*n* = 1; 5.6%); 3. patients with tension-type headache (G44.2) without medication overuse (*n* = 13; 86.7%) and with medication overuse (*n* = 2; 13.3%); as well as 4. patients with a combination of migraine and tension-type headache without (*n* = 52; 92.9%) and with (*n* = 4; 7.1%) medication overuse.

The mean number of days with a headache in the last three months before therapy began was M = 48.7 (SD 27.3), and an average of M = 24.1 days were migraine days (SD 16.2). Thus, on average, more than half of the days in a month were affected by a headache, and the mean number of days with a migraine per month was 8, which fulfills the definition of chronic migraines [8]. Patients claimed to have taken medication for their headaches in the last three months before the start of therapy on M = 26.4 days (SD 20.5), whereas patients being classified as chronic migraineurs had the highest number of medication days in the last three months (M = 30.7, SD 22.5) at t1. A percentage of 48.1% (*n* = 51) of the whole sample was treated with pain prophylaxis medication before therapy started, looking specifically at the subgroups, the highest percentage of prophylactic treatment (64.7%) was evident in chronic migraineurs as expected by applying the guidelines. The average severity of pain on a scale ranging from 0 to 10 in the last 4 weeks before the start of therapy was M = 6.0 (SD 1.8) for (self-classified) migraines and M = 4.9 (SD 1.7) for (self-classified) tension-type headaches. Headache-subgroup specific demographic and clinical characteristics of the sample based on the medical diagnoses classified by the doctors are displayed in a table in Additional File 1.

### Intervention

Interdisciplinary treatment, which is defined by the German statutory health insurance as “multi-modal pain therapy,” requires a) interdisciplinary diagnostics by at least two disciplines and b) the application of at least three therapeutic approaches out of: psychotherapy, physiotherapy, relaxation methods, occupational therapy, medical training, sensorimotor training, work place training, therapies from the area of art or other exercise therapies [28].

The interdisciplinary treatment for headache patients applied here was as a semi-inpatient, group treatment with different therapeutic approaches (e.g., physical therapy, psychoeducation, relaxation techniques, psychotherapeutic content, pharmacological treatment). Groups consisted of 8 patients on average and lasted for 2 weeks for 6 to 8 h per day for a total of 5 days per week.

The program included:

- A physical exercise program (every day): general fitness exercises, muscle strengthening exercises, stretching
- Education (every day): anatomy, pain, medication, physical and mental coping strategies
- Relaxation training (every day): progressive muscle relaxation, applied relaxation
- Optional therapy-related drug treatment or physiotherapy
- Individual physical counseling (once a week): vicious circle of pain, realistic goal determination, individually based medical therapy
- Individual psychological counseling (once a week): cognitive-behavioral intervention, treatment of individual emotional distress, mindfulness-based techniques

Following, the process how patients were selected and entered interdisciplinary treatment: patients who contacted the interdisciplinary treatment center were first sent a questionnaire. Different physicians evaluating the questionnaires made an expert-based decision concerning possible suitability for semi-inpatient interdisciplinary treatment. Then, those pre-chosen patients were screened in person with an extensive assessment by a team of a physician and a psychologist, following the guidelines for the indication of pain management programs [28]. The final decision about participation was made by the patient (shared decision making). We have described the treatment paths and selection processes including predictors for entering interdisciplinary treatment elsewhere [29].

### Instruments

In addition to the assessment of demographic variables and variables for measuring pain, the following variables for measuring health behavior and the use of health services were assessed at the start of therapy and at the 12-month follow-up:

A) Exercise/Training (aerobic and athletic):
  1. How often do you engage in endurance training (at least 30 min.) per week? (never/once/twice/three times or more)
  2. How often do you carry out exercises for muscle strengthening or stretching per week? (never/once/twice/three times or more)
B) Relaxation:
  1. How often do you use relaxation techniques (at least 10 min.) per week? (never/once/twice/three times or more)
  2. How often do you use a TENS device per week? (never/once/twice/three times or more)
    (TENS = Transcutaneous Electrical Nerve Stimulation)
  3. Do you use psychological coping strategies to deal with pain? (yes/no)
    If yes, which? (Distraction/Break-taking/Planned time slots for pleasant activities/Setting boundaries [e.g. delegation, saying no]/Giving priority to one’s own health and needs/Other strategies)
  4. Do you use mindfulness-based training/techniques? (yes/no)
C) Use of medical health services
  1. How many times did you visit your general practitioner in the last year for a problem related to pain (including appointments for prescriptions)? (0/1–4/5–8/9–12/13 or more)
  2. How many times did you visit a medical specialist for a problem related to pain in the last year (including appointments for prescriptions)? (0/1–4/5–8/9–12/13 or more)
  3. Did you make use of outpatient psychotherapy in the last year? (yes/no)
D) Use of non-medical health services
  1. Did you use physiotherapy for your headaches in the last year (e.g., manual therapy, remedial gymnastics)? (yes/no)
    If yes, how many appointments did you have? (1-6/7-12/13-18/19 or more)
  2. How many pain-related appointments did you have with a non-medical (CAM) practitioner in the last year? (0/1–4/5–8/9–12/13 or more)
    (CAM = Complementary and Alternative Medicine)
  3. How many pain-related acupuncture appointments did you have in the last year? (0/1–10/11–20/21 or more)

Additionally, patients’ direct pain-related expenses were assessed: “Approximately how much money did you spend privately in the last 3 months because of your headaches (including patient co-payments for medications)?”.

Outcome variable: The definition of success was a reduction in the number of pain days at the 12-month follow-up compared with the start of therapy. This was computed as a difference score between the pain-days assessment variable “On how many days in the last 3 months did you have a headache?” from the two assessment points. A dichotomous variable “Reduction in pain days (yes/no)” was built, with all cases with the same number of days with a headache or more days with a headache at follow-up being classified as “no” and all others as “yes.” The outcome was assessed with the above-mentioned structured question, but the patients had also kept a headache diary. They were asked to use their diary data when answering the outcome question.

### Statistical analysis

To investigate the first research question, we computed descriptive and inferential statistics. Depending on the scaling of the variables, the Wilcoxon rank-sum test (for dependent samples) or the McNemar test (for dichotomous scaled items) was used to explore changes in the sample of headache patients who received an interdisciplinary treatment between the start of therapy and one year after treatment. As a sensitivity analysis, the Benjamini–Hochberg correction method [30] for multiple testing was applied to account for the accumulation of the alpha error [31]. The alternative *p*-values are reported in the Results section.

To investigate the second research question, a binary logistic regression with the dichotomous variable “reduction in pain days” (yes = 1/no = 0) as the outcome was calculated. The twelve variables assessing health behavior and the use of health services at the 12-month follow-up were used as dichotomous or metric rescaled predictors to explore whether the successful treatment of headache symptoms was associated with health behaviors, skills taught in interdisciplinary therapy, or outpatient medical and non-medical care use. According to Vittinghof et al. (2007), the sample size was sufficient for the number of predictors we explored [32]. As a sensitivity analysis, the binary logistic regression was alternatively carried out with the more conservative dichotomous outcome “at least a 50% reduction in pain days” (yes/no). Statistical analyses were computed with SPSS 24.

## Results

### Status and changes in health behavior and the use of health services one year after treatment in headache patients receiving an interdisciplinary treatment.

A) Exercise/Training (aerobic and athletic)
  The frequency of (aerobic) endurance training (at least 30 min. per week) in the sample did not show a statistically significant change between the start of therapy and 12 months later (*p* = 0.071). Descriptively (Table 1), the percentage of patients who did not exercise at all went down from about one third (35.8%, *n* = 38) at the beginning to about one fifth (21.8%, *n* = 22) at the end, whereas the share of people who exercised intensely at least three times a week rose to about one quarter of the sample (24.8%, *n* = 25).

**Table 1:** Endurance (aerobic) and athletic exercise per week in the sample at the two measurement points

Frequency of training per week	Aerobic training **n (%) T1**	Aerobic training **n (%) T2**	Athletic training n (%) T1	Athletic training n (%) T2
Never	38 (35.8)	22 (21.8)	38 (35.8)	14 (13.9)
Once	28 (26.4)	35 (34.7)	37 (34.9)	25 (24.8)
Twice	22 (20.8)	19 (18.8)	19 (17.9)	33 (32.7)
3 times or more	18 (17.0)	25 (24.8)	12 (11.3)	29 (28.7)
  The picture for athletic sports (muscle strengthening and stretching) was different: the frequency changed significantly in the sample receiving the interdisciplinary treatment (*p* < 0.001). The median rose from 1 to 2 athletic training sessions per week when comparing the start of treatment with 12 months later. The share of patients not exercising at all was almost two thirds lower at the 12-month follow-up (Table 1), whereas more than 60% of the chronic headache patients receiving an interdisciplinary treatment exercised two or more times per week even 12 months after therapy. This portion about doubled in comparison with the start of therapy. Looking closer at the proportion of people who changed their athletic exercise behavior in the group of “non-exercisers at t1”: 32% remained stable in the group, while 29% changed their behavior to exercising once, 24% to twice and 15% to three times or more a week. Thus, about two thirds of them were training at a higher frequency in the long term.
B) Relaxation
  The use of relaxation techniques for at least 10 min per week was rather low at the time when interdisciplinary treatment began, and almost half of the sample (46.2%, *n* = 49) was not using such techniques at all. At the 12-month follow-up, the majority of the sample was still regularly using the relaxation techniques they had been taught; only one fifth of the headache patients (20.8%, *n* = 21) did not use relaxation techniques at least once a week. The portion of intensive regular users (3 times or more/week) was about twice as high at T2 (39.6%, *n* = 40) than before therapy began (18.9%, *n* = 20). The difference in the frequency of the use of relaxation techniques between the two measurement points was significant (*p* < 0.001).
  We explored how often patients used a transcutaneous electrical nerve stimulation (TENS) device per week. Before interdisciplinary treatment, the majority of the chronic headache patients (89.6%, *n* = 95) did not make use of such a device. The percentage of users rose after they participated in the treatment (from 10.4% (*n* = 11) to 25.7% (*n* = 26)), which represents a statistically significant change (*p* = 0.008). The number of patients who claimed to use psychological coping strategies to deal with pain was significantly higher one year after therapy (89.1%, *n* = 90) than before interdisciplinary treatment (53.8%, *n* = 57) (χ^2^ = 28.658; *p* < 0.001). Figure 1 shows which strategies were preferred. There was an obvious change between the two measurement points in some strategies (e.g., less “distraction” but more “giving priority to one’s own health and needs”). The use of mindfulness-based training/techniques was significantly different between before treatment and 12 months later: 17.9% (*n* = 19) claimed to use it when therapy began, and 56.4% (*n* = 57) at the 12-month follow-up (χ^2^ = 33.581; *p* < 0.001).

**Fig. 1:**
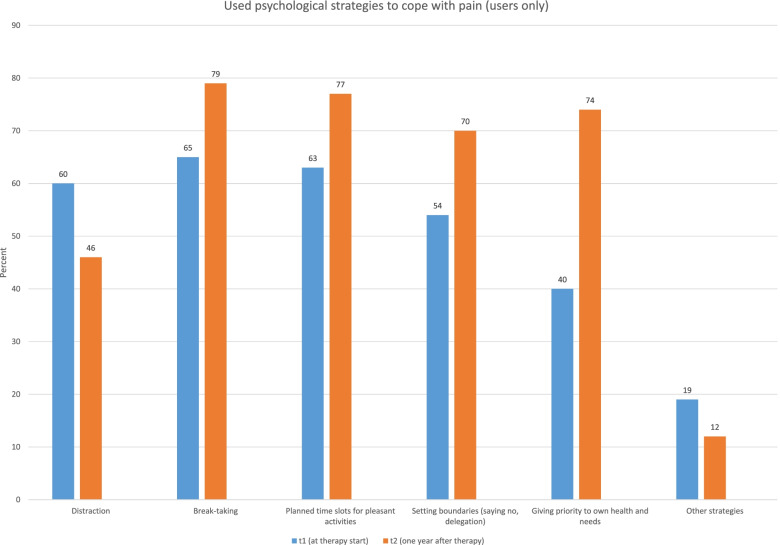
Type of coping strategies used to deal with pain compared between the two assessment points
C) Use of medical health services
  Medical health service use was explored in terms of the number of pain-related visits to the general practitioner, the number of pain-related visits to (different) medical specialists, and the use of outpatient psychotherapy with a certified medical or psychological psychotherapist during the past year.
  The number of pain-related visits to a general practitioner remained relatively constant (*p* = 0.495) with a slight downward shift in the category of “heavy users” who made 13 or more visits per year (Table 2). Almost all (91.5% (*n* = 97)/94.1% (*n* = 95)) chronic headache patients made pain-related general practitioner visits.

**Table 2:** Chronic headache patients’ number of pain-related visits to general practitioners (GPs) and medical specialists per year

**Number of visits per year (including appointments for prescriptions)**	**GP** visits **n (%) T1**	**GP** visits **n (%) T2**	**Medical specialist** visits **n (%) T1**	**Medical specialist visits n (%) T2**
None	9 (8.5)	6 (5.9)	9 (8.5)	18 (17.8)
One to Four	34 (32.1)	40 (39.6)	50 (47.2)	41 (40.6)
Five to Eight	25 (23.6)	21 (20.8)	19 (17.9)	23 (22.8)
Nine to Twelve	19 (17.9)	19 (18.8)	20 (18.9)	6 (5.9)
Thirteen or more	19 (17.9)	15 (14.9)	8 (7.5)	13 (12.9)
  Before interdisciplinary treatment began, more than 90% of the chronic headache patients had visited pain-related medical specialists, but the percentage of users in general was lower at the 12-month follow-up (82.2%, *n* = 83) (Table 2). However, the number of patients categorized as “heavy users” with 13 or more pain-related specialist visits per year was higher one year after interdisciplinary treatment (7.5%, *n* = 8 vs. 12.9%, *n* = 13). However, the difference in the pattern of use was not statistically significant (*p* = 0.134). The proportion of chronic headache patients who used outpatient psychotherapy in the past year was different but did not quite meet the threshold for statistical significance (χ^2^ = 3.692; *p* = 0.055): One fourth (25.5%, *n* = 27) was enrolled in outpatient psychotherapy before interdisciplinary treatment began, whereas more patients (39.6%, *n* = 40) claimed that they used this service at the 12-month follow-up.
D) Use of non-medical health services
  The percentage of patients who used physiotherapy for their headaches (e.g. manual therapy, remedial gymnastics) during the past year was about half of the sample with a slightly (but non-significantly) higher share before interdisciplinary treatment (57.5% (*n* = 61) at T1; 48.5% (*n* = 49) at the 12-month follow-up) (χ^2^ = 1.441; *p* = 0.230). Those who used physiotherapy (*n* = 61 (T1); *n* = 49 (T2)) had usually been given one (29.5% of users (*n* = 18) at T1; 40.8% of users (*n* = 20) at T2) or two prescriptions (32.8% of users (*n* = 20) (T1); 30.6% of users (*n* = 15) (T2)). In Germany’s statutory health insurance, one prescription for a non-medical therapy (physiotherapy counts as that) comprises six applications/appointments. The utilization pattern changed significantly between the two timepoints (*p* = 0.038). Especially the portion of intensive “heavy users” who received at least four prescriptions and thus visited physiotherapists 24 or more times per year decreased from 26.2% (*n* = 16) to 16.3% (*n* = 8).
  We also explored the use of treatments with less scientific evidence of their effectiveness in the treatment of chronic headache disorders. Representing alternative medicine, patients were asked how many pain-related appointments with a non-medical (complementary and alternative medicine) practitioner they had in the last year. The proportion using this non-medical health service, which is usually not covered by the German statutory health insurance, decreased from about one quarter (*n* = 27) (before interdisciplinary treatment) to 15% (*n* = 15) (12-month follow-up) (Table 3). The decline in the use of non-evidenced-based treatment was significant (*p* = 0.031).

**Table 3:** Chronic headache patients’ number of pain-related visits to non-medical practitioners and number of pain-related acupuncture visits per year

Number of visits to non-medical practitioners per year	n (%) at T1 (therapy start)	n (%) at T2 (12-month FU)
None	79 (74.5)	86 (85.1)
One to Four	16 (15.1)	11 (10.9)
Five to Eight	5 (4.7)	1 (1.0)
Nine to Twelve	5 (4.7)	2 (2.0)
Thirteen or more	1 (.9)	1 (1.0)
**Number of pain-related acupuncture appointments per year**		
None	79 (74.5)	89 (88.1)
One to Ten	19 (17.9)	9 (8.9)
Eleven to Twenty	6 (5.7)	1 (1.0)
Twenty-one or more	2 (1.9)	2 (2.0)
  As a second type of non-medical treatment, the utilization of acupuncture was explored. This is also a health service that has to be self-paid by patients, and health insurance usually does not reimburse the costs, even though there are hints for effectiveness. Acupuncture is usually offered in packages of 10 appointments. The proportions of users in general also significantly declined from about ¼ (25.5%, *n* = 27) before interdisciplinary treatment to 1/10 (11.9%, *n* = 12) at follow-up (*p* = 0.032). Table 3 shows headache patients’ frequency of use of acupuncture at the two measurement points.
  The median of patients’ direct pain-related expenses spent privately in the last 3 months because of their headaches was 55 Euro before therapy began and 40 Euro at the 12-month follow-up, which depicts a non-significant decline (*p* = 0.076).
  As a sensitivity analysis, the Benjamini–Hochberg correction method [30] for multiple testing was applied to account for the accumulation of the alpha error. Corrected *p*-values were calculated for the 14 inferential statistical procedures. Table 4 shows the original and the corrected *p*-values. As a consequence, five health-related behavioral changes in headache patients who received the interdisciplinary treatment remained to be interpreted: a higher frequency of athletic sports, a higher use of relaxation techniques and TENS devices for relaxation purposes, and a more frequent use of psychological coping strategies and mindfulness-based techniques to deal with pain – all at the follow-up assessment.

**Table 4:** A comparison of *p*-values before and after the application of a method for correcting for multiple testing

Health behavior/health service use assessed at both time points	*p*-value uncorrected	*p*-value corrected (Benjamini–Hochberg method)
Athletic exercise	< .001***	< .001***
Relaxation techniques	< .001***	< .001***
Psychological coping strategies	< .001***	< .001***
Mindfulness-based techniques	< .001***	< .001***
TENS device	.008**	.0224*
Non-medical practitioner visits	.031*	.0640
Acupuncture visits	.032*	.0640
Frequency of physiotherapy	.038*	.0665
Psychotherapy use	.055	.0856
Endurance exercise	.071	.0967
Privately spent expenses	.076	.0967
Medical specialists visits	.134	.1563
Physiotherapy use	.230	.2477
GP visits	.495	.4950

### Association of health behaviors/health service use patterns with “successful” treatment in terms of a reduction in pain days

About half of the sample 52.8%, (*n* = 56) had a reduction in pain days at follow-up in comparison with the beginning of the therapy; 23.6% (*n* = 25) reached a 50% reduction in their pain days. The mean number of headache days in the last three months was M = 42.0 (SD: 27.5) at follow-up, which depicts an average reduction of seven pain days in comparison with the first assessment. The difference is statistically significant (t = 3.092; *p* = 0.003), with a small effect size of Cohen’s d = 0.302 (Confidence Interval (CI): 0.105—0.497).

Subgroup-specific analysis according to headache type revealed that pain days reduction was the greatest in the subgroup of chronic migraine patients respectively chronic migraineurs with additional tension-type headache (64.7% resp. 57.1% of patients in those subgroups showed less pain days at follow-up; for the combined subgroup: t (55) = 3.127; *p* = 0.001; Cohen’s d: 0.418 [0.143; 0.689]). The subgroup-specific statistics concerning pain days and their reduction is depicted in Additional File 1.

More than half of the sample (53.8%, *n* = 57) reported a reduced number of their medication days at follow-up in comparison to the first assessment. The mean number of medication days in the last three months was M = 22.5 (SD: 15.5) at follow-up, which depicts an average reduction of almost four days in comparison before treatment. The difference is statistically significant (t = 1.685; *p* = 0.047), with an effect size of 0.164 (Cohen’s d; CI: -0.028—0.355). Again, the subgroup of chronic migraine patients showed the largest effect.

The average severity of pain at follow-up was M = 5.6 (SD 2.1) for (self-classified) migraines and M = 4.1 (SD 1.6) for (self-classified) tension-type headaches. For both subgroups a statistically significant reduction of pain severity was observable for self-classified migraine (t = 1.759; *p* = 0.041; Cohen’s d = 0.171 (CI: -0.021—0.362)) and for self-classified tension-type headaches (t = 5.088; *p* < 0.001; Cohen’s d = 0.494 (CI: -0.291—0.695)). Headache-subgroup specific comparisons based on the medical diagnoses classified by the treatment suppliers of pain days, medication days and pain severity are displayed in a table in Additional File 1. There, it is obvious, that a significant reduction of pain severity was seen in the subgroup of chronic migraine patients respectively chronic migraineurs with additional tension-type headache (for the latter group: t (55) = 2.997; *p* = 0.002; Cohen’s d: 0.401 [0.126; 0.671]). At follow-up 49.1% of the patients (*n* = 52) were taken pain prophylaxis medication. The percentage was basically stable in comparison to therapy start (*p* = 1.000).

A binary logistic regression with the dichotomous outcome reduction in pain days at follow-up (yes = 1/no = 0) and 12 predictors from the areas of health behavior, medical service use, use of alternative medicine, and adjuvant therapy was carried out. A significant model with an acceptable explained variance of 25.5% (R^2^ Nagelkerke) resulted (χ^2^ (12) = 21.419; *p* = 0.045), interpretable as a small effect [33]. Existing regular physical activity in the form of muscle strengthening and stretching (athletic sports) was positively associated with a reduction in pain days 12 months after treatment (*p* = 0.012; OR: 1.96). Thus, every additional unit of athletic sports per week doubled the chance of reducing the number of pain days. The use of psychotherapy in the last 12 months was negatively associated with a reduction in pain days (*p* = 0.014; OR: 0.28). Thus, patients with major mental comorbidities needing additional outpatient treatment for such conditions have a smaller chance of reducing their number of pain days 12 months after treatment. In other words, in our study, patients seeking psychotherapy had a 257% higher chance of having an unchanged (non-reduced) pain symptom load in comparison with patients who did not have additional psychotherapeutic treatment (Table 5).

**Table 5:** Binary logistic regression with the reduction in pain days as the outcome at the 12-month follow-up

**Variables**	**Regression coefficient β**	**Standard error**	**Wald**	***p*-value**	**OR**	**95%** CI for OR **Lower Bound**	**95%** CI for OR **Upper Bound**
Endurance (aerobic) exercise per week	-.105	.222	.221	.638	.901	.583	1.392
Athletic training (muscle strengthening and stretching) per week	.672	.266	6.376	**.012**	**1.959**	1.162	3.300
Use of relaxation techniques per week	.112	.215	.271	.602	1.119	.734	1.706
TENS device use per week^a^	.116	.231	.254	.614	1.123	.715	1.765
Use of psychological coping strategies to deal with pain^c^	1.318	.756	3.041	.081	3.735	.849	16.426
Use of mindfulness-based training/techniques^c^	.075	.466	.026	.872	1.078	.433	2.685
Number of pain-related general practitioner visits per year	-.350	.212	2.743	.098	.704	.465	1.066
Number of pain-related medical specialist visits per year	-.088	.210	.177	.674	.915	.606	1.382
Use of outpatient psychotherapy in the last year^c^	-1.280	.522	6.018	**.014**	**.278**	.100	.773
Use of physiotherapy due to headache in the last year^c^	-.464	.456	1.038	.308	.629	.257	1.536
Number of pain-related non-medical (CAM) practitioner visits per year^b^	-.645	.428	2.274	.132	.525	.227	1.213
Number of pain-related acupuncture appointments per year	.607	.477	1.621	.203	1.835	.721	4.670

^a^*TENS* **T**ranscutaneous **E**lectrical **N**erve **S**timulation

^**b**^*CAM* **C**omplementary and **A**lternative **M**edicine

^c^dichotomous: 0 = no; 1 = yes

As a second sensitivity analysis, the binary logistic regression was alternatively carried out with the more conservative outcome “at least a 50% reduction in pain days” (yes/no). A significant model did not result (χ^2^ (12) = 9.861; *p* = 0.628), presumably due to the smaller number of participants fulfilling the criterion and thus less variability. However, the significant predictor “existing regular physical activity in the form of athletic sports” reported above was also (although nonsignificantly) associated with the outcome of at least a 50% reduction in pain days (*p* = 0.062).

## Discussion

The data we explored showed that a cohort of headache patients receiving an interdisciplinary treatment acquired skills, probably during treatment, that were kept up 12 months later. These skills referred to regular physical activity in the sense of athletic training (muscle strengthening and stretching) and to the use of different techniques for relaxation. Besides these skills, psychological strategies and techniques for dealing with the pain that still existed were learned and used.

A limitation of this study is the lack of a control group so that we do not know the extent to which chronic headache patients would have acquired these skills without interdisciplinary treatment. However, the literature shows that interdisciplinary (multimodal) pain therapy leads to changes in health behavior in the long term [20, 21], and it was not the aim of the study to demonstrate efficacy as another study design would have been necessary for that. However, others also described an “adherence to lifestyle modifications” in the long term after interdisciplinary treatment such that the majority of the patients implemented a change in health behavior with respect to relaxation and aerobic endurance sports [34]. In fact, the percentages of patients who kept up a new routine involving the use of relaxation techniques and engaging in endurance sports were comparable to our study: 61% [34] / 79% (this study) regularly practiced relaxation techniques, 72% [34] /78% (this study) engaged in endurance sports.

Besides the change in health-related behavior from acquiring skills, interdisciplinary treatment for chronic headache patients has been shown to be effective in lowering the burden of illness in terms of pain days or headache-related disability [26, 35–37]. Also, in our study, the average pain days were lower at the follow-up assessment, and about half of the sample (53%) reported a smaller number of pain days 12 months after treatment. On average, at follow-up, the sample fell below the definition of a chronic headache at below 15 days of headaches per month, whereas at the beginning of treatment, the definitions of chronic headaches and chronic migraines were fulfilled. The percentage of people reaching the criterion of at least a 50% reduction in the number of pain days of 24% was comparable to Gaul et al. [34], who reported that 29% of the patients who had a combination of migraines and tension-type headaches reached the goal of at least 50% reduction in pain days.

Again, the extent to which the number of pain days would have decreased without interdisciplinary treatment is unknown because of the lack of a control group. However, the literature confirms the effectiveness of the interdisciplinary treatment of headache patients in terms of decreasing the number of pain days [26, 36, 37]. Thus, the change might be largely evoked by treatment participation.

Regarding exercise training, there is evidence that especially high-intensity interval training is somewhat superior to moderate continuous exercise programs for migraine patients [38], however the findings apply for episodic migraineurs. A recent meta-analysis supported the beneficial effects of aerobic exercise for migraine patients in terms of pain frequency [39]. Another systematic review reported the encouraging effects of strengthening exercises for neck pain patients [40], also supported by a Cochrane review [41]. Thus, aerobic and athletic exercise is associated with treatment success in pain patients in general and also specifically for headache patients [34]. A recent meta-analysis also revealed the optimal dose–response relationship in stabilization exercises for pain patients [42]. In our study with headache patients, regular physical activity in terms of athletic sports (muscle strengthening and stretching) predicted treatment success measured in a reduction in pain days. The study results showed skill growth/development over the two measurement points in exactly that form of physical activity next to relaxation skills and psychological coping strategies. However, only physical activity was associated with the definition of treatment success but not relaxation skills or coping strategies. This result consequently underlines the importance of teaching exercise skills and implementing those skills as routines into the everyday lives of headache patients. Thus, our data suggest that a physical activity module (with athletic training components) is a key element in interdisciplinary treatment concepts.

The cohort of headache patients explored in our study was comparable in age with other studies for example those included in a recent meta-analysis [39] or in another German tertiary headache center [34]. However, other groups of interdisciplinary treated chronic pain patients in interdisciplinary pain centers seem to be older [e.g. 43]. Age (and together with younger age, lower physical comorbidity and impairment) might be a factor in motivation and ability for engagement in athletic sports over a longer time.

The frequency of the use of “alternative medicine,” such as acupuncture or CAM treatments, was comparable to an Austrian study before patients were enrolled in interdisciplinary treatment. There, about ¼ of the headache patients reported using acupuncture (28.1%) and homeopathy (24.6%) before therapy began in an interdisciplinary treatment center [44]. Comparable numbers were assessed in this study before interdisciplinary treatment began (25.5% acupuncture, 25.5% complementary and alternative medicine). Although not statistically significant after the *p*-values were corrected for multiple comparisons, there was a trend (i.e., significant with uncorrected *p*-values) that the use of questionable evidence-based treatments (e.g., non-medical practitioner visits) decreased after headache patients participated in an interdisciplinary treatment. As expected, the frequency of pain-associated GP visits or visits to medical specialists remained unchanged. This result was expected and reflected the organized transition from semi-inpatient to outpatient medical care. The smooth transition cannot be taken for granted as researchers have described the problem of continuity of health care for headache patients [45]. The boundary point between the treatment settings in pain therapy responsible for continuing the therapeutic regime set up in the interdisciplinary treatment program seems to work at least in the sense of the application and utilization of care in an outpatient medical health care system. Supporting our results, Brilla et al. [46] showed that headache patients treated in a headache clinic require a large amount of outpatient remote care, second only to neurology patients.

Our results suggest that the additional use of psychotherapy was negatively associated with a reduction in the number of pain days at follow-up. This finding should not be interpreted to mean that psychotherapeutic treatment causes impairments, but it rather underlines the larger burden of illness in highly comorbid patients. Mental comorbidities in chronic pain patients are common [47, 48] and are associated with lower therapeutic success [49, 50] in chronic pain treatment. Thus, our results support those found in the literature [49, 50]. Chronic pain (here, headache) patients with mental comorbidities pose a special therapeutic challenge. A focus on mental comorbidities should be included as an additional module in interdisciplinary treatment for those patients who are affected, and modularized developments already exist in interdisciplinary therapy [50]. This additional integrated psychotherapeutic approach by pain specialists in addition to outpatient psychotherapy could help produce a desired pain-specific outcome, such as fewer pain days or less medication use.

### Limitations

The study was carried out as an evaluation of routine data from a treated cohort of chronic headache patients. Therefore, all of the individuals who were analyzed received interdisciplinary treatment, and a comparison with untreated individuals was not possible. Thus, the data do not allow classic interpretations of the efficacy of interdisciplinary treatment. Estimations of the amount of change in health behavior and health service use in chronic headache patients without interdisciplinary treatment is not possible.

Second, the sample was small even though data of an enrolment period of five years were included. The small sample size might have led to a lack of power to detect changes between the two time points. For example, there have been statistical trends in the change in the use of treatments involving acupuncture or complementary/alternative medicine that would have been statistically significant in a larger sample with a larger number of participants who used such treatments. Consequently, existing clinically relevant changes appear and are interpreted as statistically non-significant. The small sample is also a disadvantage when analyzing different “success” outcomes. Setting more strict boundaries in defining outcomes implies that fewer patients are fulfilling the criteria, which again consequently results in a lack of power and a lack of variability in those fulfilling the success category. Thus, we will need to repeat such analyses with different outcomes (e.g., “at least a 50% reduction in the number of pain days”; “progression from chronic to episodic pain”) with a larger sample after more patients are enrolled in the future.

Third, routine data were assessed from one interdisciplinary pain treatment center. Whether the results are generalizable to other centers can be discussed. However, the treatment concept is manualized and implemented in all German semi-inpatient/inpatient treatment centers, and the outcome assessment is standardized in those using the evaluation module from the German Pain Questionnaire [51]. In a previous publication we analyzed the predictors for entering interdisciplinary treatment in the same treatment center where we acquired the data for this manuscript [29]. Main predictors for inclusion were being employed, having a younger age, not having an application for retirement in consideration, having a higher affective experience of pain, having a higher self-rating of owns pain as treatable and having more often participated in at least one pain-associated rehabilitation treatment in the past.

Fourth, comparison data for health care utilization are largely unavailable, especially regarding headache patients undergoing an interdisciplinary treatment. There has been one representative German study exploring the frequency of GP use in the general population with self-reported headache. Compared with that, the patients in our sample had more pain-related GP visits on a regular basis. However, Müller et al. assessed the general population reporting headaches, not chronic headache patients, which was the highly burdened sample undergoing interdisciplinary treatment we assessed [25]. Likewise, findings were reported by an Italian study that assessed pharmacy clients with headaches [14] in which about 1/3 of self-reported migraine-affected patients and almost half of the self-reported headache-affected patients had not contacted GPs or medical specialists for help with their pain.

## Conclusion

Chronic headache patients who participated in an intensive multimodal interdisciplinary treatment on average acquired skills that were available in the long term and implemented into their daily routines. Such skills concern the acquisition and use of relaxation techniques, the use of psychological strategies to deal with still existing chronic pain, and regular physical activity in terms of athletic exercise. Of those health behavior skills, a regular engagement in athletic sports was positively associated with a decrease in (headache) pain days in the long term. Thus, interdisciplinary treatment concepts should not be applied without a physical activity module.

## Supplementary Information

**Additional file 1.**

## Data Availability

The data sets used and analyzed in the current study are available from the corresponding author upon reasonable request.

## Abbreviations

CAM: Complementary and alternative medicine
GP: General practitioner
IASP: International Association for the Study of Pain
TENS: Transcutaneous Electrical Nerve Stimulation
WHO: World Health Organization
YLDs: Years of life lived with disability
