# Supplementary material for: Chronic headache patients’ health behavior and health service use 12 months after interdisciplinary treatment – what do they keep in their daily routines?
Source: BMC Neurol. 2022 Apr 21;22:149. doi: 10.1186/s12883-022-02646-w (PMC9022266; doi:10.1186/s12883-022-02646-w)
Supplement: Supplementary file 1 — Additional file 1. [file 12883_2022_2646_MOESM1_ESM.docx]

Additional File 1

Subgroup-specific characteristics of the sample before start of therapy and subgroup-specific data in outcome variables

| Variable | Chronic migraine (n = 17)  n (%) / M(SD) | Episodic migraine (n = 18)  n (%) / M(SD) | Tension-type headache (n = 15)  n (%) / M(SD) | Combination of migraine and tension-type headache (n = 56)  n (%) / M(SD) |
| --- | --- | --- | --- | --- |
| *Demographics* |  |  |  |  |
| Age (years) | 42.5 (10.2) | 39.6 (11.6) | 37.8 (13.7) | 36.7 (13.3) |
| Sex (female) | 17 (100) | 16 (88.9) | 13 (86.7) | 51 (91.1) |
| Marital status  (married)  (single) | 8 (47.1)  6 (35.3) | 6 (33.3)  10 (55.6) | 7 (46.7)  7 (46.7) | 21 (37.5)  31 (55.4) |
| Living alone in the household | 2 (11.8) | 5 (27.8) | 2 (13.3) | 13 (23.2) |
| Migration background (no) | 16 (94.1) | 17 (94.4) | 13 (85.7) | 48 (86.7) |
| Education  (10 years or less)  (university degree) | 3 (17.6)  8 (47.1) | 1 (5.6)  7 (38.9) | 3 (20.0)  5 (33.3) | 7 (12.5)  17 (30.4) |
| Active workforce/education (yes) | 14 (82.4) | 14 (77.8) | 12 (80.0) | 44 (78.6) |
| Accepted degree of disability (yes) | 3 (17.6) | 4 (22.2) | 2 (13.3) | 3 (5.4) |
| Permanent sick leave (yes) | 2 (11.8) | 1 (5.6) | 1 (6.7) | 3 (5.4) |
| *Clinical characteristics* |  |  |  |  |
| Duration of headache condition  (1 -2 years)  (2 – 5 years)  (more than 5 years) | 0 (0.0)  1 (5.9)  16 (94.1) | 1 (5.6)  0 (0.0)  17 (94.4) | 3 (20.0)  3 (20.0)  7 (46.7) | 3 (5.4)  11 (19.6)  39 (69.6) |
| Pain characteristics  (Permanent with slight deviation)  (Permanent with large deviation)  (Attacks without pain in-between)  (Attacks with pain in-between) | 3 (17.6)  1 (5.9)  7 (41.2)  6 (35.3) | 1 (5.6)  0 (0.0)  14 (77.8)  3 (16.7) | 6 (40.0)  4 (26.7)  2 (13.3)  2 (13.3) | 1 (1.8)  9 (16.1)  27 (48.2)  18 (32.1) |
| Frequency of attacks  (answers only if attacks were stated in pain characteristics)  (% of subgroup with attacks)  (daily)  (Once/several times a week)  (Once/several times a month) | n = 13  0 (0.0)  9 (69.2)  4 (30.8) | n = 17  0 (0.0)  12 (70.6)  5 (30.4) | n = 4  1 (6.7)  2 (50.0)  1 (25.0) | n = 45  2 (4.4)  29 (64.5)  14 (31.1) |
| Duration of attacks  (answers only if attacks were stated in pain characteristics)  (% of subgroup with attacks)  (several minutes)  (several hours)  (up to three days)  (ore than 3 days) | 0 (0.0)  1 (7.7)  10 (76.9)  2 (15.4) | 0 (0.0)  8 (47.1)  7 (41.2)  2 (11.8) | 0 (0.0)  3 (75.0)  0 (0.0)  1 (25.0) | 1 (2.2)  16 (35.6)  24 (53.3)  4 (8.9) |
| *Outcome Variables* |  |  |  |  |
| Pain days last 3 months (before therapy start) | 46.5 (21.3) | 24.5 (9.7) | 70.7 (27.8) | 51.3 (27.1)***** |
| Pain days last 3 months (follow-up) | 34.0 (20.8) | 25.4 (15.2) | 68.3 (32.0) | 42.4 (26.1)***** |
| t-test for dependent samples  (t (df) = …; p = …) | (t (16) = 1.738; p = .051) | (t (17) = -.282; p = .391) | (t (14) = .329; p = .373) | (t (55) = 3.127; **p = .001**)  **Cohen’s d: .418** [.143; .689] |
| Medication days last 3 months  (before therapy start) | 30.7 (22.5) | 21.4 (9.7) | 22.4 (24.5) | 27.7 (21.2) |
| Medication days last 3 months  (follow-up) | 22.8 (13.4) | 23.6 (13.9) | 21.5 (29.0) | 22.2 (11.4) |
| t-test for dependent samples  (t (df) = …; p = …) | (t (16) = 1.455; p = .083) | (t (17) = -.762; p = .228) | (t (14) = .077; p = .470) | (t (55) = 2.060; p = .022)  Cohen’s d: .275 [.007; .541] |
| Pain severity last 4 weeks (0 – 10)  (before therapy start) | 6.1 (1.7) | 5.2 (1.7) | 4.9 (1.6) | 5.1 (1.9) ***** |
| Pain severity last 4 weeks (0 – 10)  (follow-up) | 5.1 (2.4) | 4.9 (1.9) | 4.4 (1.4) | 4.3 (1.8) ***** |
| t-test for dependent samples  (t (df) = …; p = …) | (t (16) = 2.104; p = .026) | (t (17) = .687; p = .251) | (t (14) = 1.628; p = .063) | (t (55) = 2.997; **p = .002**)  **Cohen’s d: .401** [.126; .671] |
| n/percentage of the subsample with reduced pain days at follow-up | 11 (64.7) | 8 (44.4) | 5 (33.3) | 32 (57.1) |
| n/percentage of the subsample with at least 50%-less pain days at follow-up | 6 (35.3) | 4 (22.2) | 3 (20.0) | 12 (21.4) |
| n/percentage of the subsample with reduced medication days at follow-up | 12 (70.6) | 7 (38.9) | 10 (66.7) | 28 (50.0) |

Level of significance in t-test for dependent samples: p =.05; Bonferroni-corrected significance level: p = .004 – marked with *****
